# Supplementary material for: Knowledge of Polish Nurses About Sepsis Based on Validated Questionnaire: A Multi-Site Cross-Sectional Study
Source: Nurs Rep. 2025 May 30;15(6):195. doi: 10.3390/nursrep15060195 (PMC12196121; doi:10.3390/nursrep15060195)
Supplement: Supplementary file 1 [file nursrep-15-00195-s001.zip › Supplementary file S1.pdf]

Supplementary file S1.

Comparison of the level of general knowledge, its dimensions, attitude and self-assessment of knowledge regarding the level of voluntary postgraduate education

Table 1.1.sf. Comparison of general knowledge, its dimensions, attitude, and self-assessed knowledge by participation in internal workplace training.

	No (n = 170)		Yes (n = 123)				95% *CI*		
	M	*SD*	*M*	*SD*	*t*	*p*	*LL*	*UL*	*d*
General knowledge	9.54	3.51	9.42	3.21	0.28	0.779	-0.68	0.90	0.03
Factor 1	5.73	2.64	5.70	2.43	0.10	0.920	-0.56	0.63	0.01
Factor 2	3.81	1.63	3.72	1.79	0.41	0.683	-0.31	0.48	0.05
Attitude	21.25	4.79	22.19	4.16	-1.74	0.083	-1.99	0.12	0.21
Self-assessment knowledge	3.14	1.08	3.24	1.01	-0.76	0.449	-0.34	0.15	0.09

M – mean; SD – standard deviation; t – t-statistic; p – significance level; CI – confidence interval; LL – lower limit; UL – upper limit; d – Cohen's d effect size.

Table 1.2.sf. Comparison of general knowledge, its dimensions, attitude, and self-assessed knowledge by participation in specialization training.

	No (n = 180)		Yes (n = 113)				95% *CI*		
	M	*SD*	*M*	*SD*	*t*	*p*	*LL*	*UL*	*d*
General knowledge	9.44	3.43	9.56	3.30	-0.28	0.781	-0.91	0.69	0.03
Factor 1	5.70	2.67	5.74	2.36	-0.14	0.888	-0.65	0.56	0.02
Factor 2	3.74	1.62	3.81	1.81	-0.34	0.733	-0.47	0.33	0.04
Attitude	20.89	4.52	22.84	4.35	-3.64	<0.001	-3.00	-0.89	0.44
Self-assessment knowledge	3.11	1.03	3.30	1.08	-1.55	0.122	-0.44	0.05	0.19

M – mean; SD – standard deviation; t – t-statistic; p – significance level; CI – confidence interval; LL – lower limit; UL – upper limit; d – Cohen's d effect size.

Table 1.3.sf. Comparison of general knowledge, its dimensions, attitude, and self-assessed knowledge by participation in qualification courses.

	No (n = 140)		Yes (n = 153)				95% *CI*		
	M	*SD*	*M*	*SD*	*t*	*p*	*LL*	*UL*	*d*
General knowledge	9.65	3.69	9.34	3.07	0.78	0.437	-0.47	1.09	0.09
Factor 1	5.90	2.66	5.55	2.44	1.18	0.240	-0.24	0.94	0.14
Factor 2	3.75	1.75	3.79	1.65	-0.21	0.837	-0.43	0.35	0.02
Attitude	20.93	4.79	22.30	4.22	-2.59	0.010	-2.41	-0.33	0.30
Self-assessment knowledge	3.10	1.05	3.25	1.05	-1.26	0.209	-0.40	0.09	0.15

M – mean; SD – standard deviation; t – t-statistic; p – significance level; CI – confidence interval; LL – lower limit; UL – upper limit; d – Cohen's d effect size.

Tabela 1.4.sf. Comparison of general knowledge, its dimensions, attitude, and self-assessed knowledge by participation in specialized courses.

	No (n = 140)		Yes (n = 153)				95% *CI*		
	M	*SD*	*M*	*SD*	*t*	*p*	*LL*	*UL*	*d*
General knowledge	9.22	3.30	9.73	3.45	-1.29	0.197	-1.29	0.27	0.15
Factor 1	5.55	2.50	5.87	2.59	-1.07	0.285	-0.91	0.27	0.13
Factor 2	3.67	1.71	3.86	1.69	-0.96	0.336	-0.58	0.20	0.11
Attitude	20.68	4.53	22.53	4.39	-3.55	<0.001	-2.88	-0.82	0.41
Self-assessment knowledge	2.98	1.06	3.37	1.02	-3.20	0.002	-0.63	-0.15	0.37

M – mean; SD – standard deviation; t – t-statistic; p – significance level; CI – confidence interval; LL – lower limit; UL – upper limit; d – Cohen's d effect size.

Table 1.5.sf. Comparison of general knowledge, its dimensions, attitude, and self-assessed knowledge by participation in other courses or training activities.

	No (n = 268)		Yes (n = 25)				95% *CI*		
	M	*SD*	*M*	*SD*	*t*	*p*	*LL*	*UL*	*d*
General knowledge	9.51	3.36	9.20	3.64	0.45	0.657	-1.08	1.71	0.09
Factor 1	5.73	2.56	5.60	2.50	0.24	0.811	-0.92	1.18	0.05
Factor 2	3.79	1.65	3.60	2.12	0.43	0.671	-0.71	1.08	0.11
Attitude	21.58	4.64	22.36	3.37	-1.07	0.292	-2.27	0.70	0.17
Self-assessment knowledge	3.16	1.07	3.44	0.77	-1.70	0.099	-0.62	0.06	0.27

M – mean; SD – standard deviation; t – t-statistic; p – significance level; CI – confidence interval; LL – lower limit; UL – upper limit; d – Cohen's d effect size.

Table 1.6.sf. Comparison of general knowledge, its dimensions, attitude, and self-assessed knowledge based on the lack of participation in qualification-enhancing courses or training.

	No (n = 254)		Yes (n = 39)				95% *CI*		
	M	*SD*	*M*	*SD*	*t*	*p*	*LL*	*UL*	*d*
General knowledge	9.57	3.42	8.95	3.09	1.07	0.285	-0.52	1.77	0.18
Factor 1	5.78	2.57	5.28	2.39	1.14	0.254	-0.36	1.36	0.20
Factor 2	3.79	1.68	3.67	1.80	0.41	0.680	-0.45	0.70	0.07
Attitude	22.13	4.27	18.51	5.07	4.79	<0.001	2.13	5.10	0.82
Self-assessment knowledge	3.26	1.03	2.67	1.08	3.33	0.001	0.24	0.94	0.57

M – mean; SD – standard deviation; t – t-statistic; p – significance level; CI – confidence interval; LL – lower limit; UL – upper limit; d – Cohen’s d effect size.
