# Supplementary material for: Knowledge of Polish Nurses About Sepsis Based on Validated Questionnaire: A Multi-Site Cross-Sectional Study
Source: Nurs Rep. 2025 May 30;15(6):195. doi: 10.3390/nursrep15060195 (PMC12196121; doi:10.3390/nursrep15060195)
Supplement: Supplementary file 1 [file nursrep-15-00195-s001.zip › Supplementary file S2.pdf]

Supplementary file S2.
Comparison of the level of general knowledge, its dimensions, attitude, and self-assessment of knowledge regarding the workplace setting.

Table 2.1.sf. Comparison of general knowledge, its dimensions, attitude, and self-assessed knowledge between nurses working and not working in the Department of Anesthesiology and Intensive Care.

	No (n = 243)		Yes (n = 50)				95% *CI*		
	M	*SD*	*M*	*SD*	*t*	*p*	*LL*	*UL*	*d*
General knowledge	9.10	3.39	11.38	2.61	-5.32	<0.001	-3.13	-1.43	0.70
Factor 1	5.49	2.58	6.82	2.07	-3.96	<0.001	-2.00	-0.66	0.53
Factor 2	3.61	1.72	4.56	1.30	-4.44	<0.001	-1.38	-0.53	0.57
Attitude	21.51	4.64	22.28	4.08	-1.18	0.241	-2.06	0.53	0.17
Self-assessment knowledge	3.18	1.06	3.18	1.02	0.01	0.995	-0.32	0.32	0.00

M – mean; SD – standard deviation; t – t-statistic; p – significance level; CI – confidence interval; LL – lower limit; UL – upper limit; d – Cohen's d effect size.

Table 2.2.sf. Comparison of general knowledge, its dimensions, attitude, and self-assessed knowledge between nurses working and not working in the Emergency Ward.

	No (n = 276)		Yes (n = 17)				95% *CI*		
	M	*SD*	*M*	*SD*	*t*	*p*	*LL*	*UL*	*d*
General knowledge	9.35	3.33	11.71	3.46	-2.82	0.005	-4.00	-0.71	0.71
Factor 1	5.64	2.50	7.00	3.06	-2.15	0.032	-2.61	-0.12	0.54
Factor 2	3.71	1.70	4.71	1.31	-2.97	0.008	-1.69	-0.29	0.59
Attitude	21.56	4.62	23.06	2.97	-1.95	0.065	-3.11	0.10	0.33
Self-assessment knowledge	3.17	1.06	3.29	0.85	-0.46	0.648	-0.64	0.40	0.11

M – mean; SD – standard deviation; t – t-statistic; p – significance level; CI – confidence interval; LL – lower limit; UL – upper limit; d – Cohen's d effect size.

Table 2.3.sf. Comparison of general knowledge, its dimensions, attitude, and self-assessed knowledge between nurses working and not working in Primary Health Care.

	No (n = 250)		Yes (n = 43)				95% *CI*		
	M	*SD*	*M*	*SD*	*t*	*p*	*LL*	*UL*	*d*
General knowledge	9.61	3.37	8.77	3.37	1.52	0.130	-0.25	1.94	0.25
Factor 1	5.82	2.55	5.09	2.47	1.74	0.083	-0.09	1.56	0.29
Factor 2	3.79	1.66	3.67	1.92	0.41	0.686	-0.44	0.67	0.07
Attitude	21.58	4.51	22.00	4.80	-0.55	0.580	-1.90	1.06	0.09
Self-assessment knowledge	3.16	1.05	3.33	1.04	-0.98	0.330	-0.51	0.17	0.16

M – mean; SD – standard deviation; t – t-statistic; p – significance level; CI – confidence interval; LL – lower limit; UL – upper limit; d – Cohen's d effect size.

Table 2.4.sf. Comparison of general knowledge, its dimensions, attitude, and self-assessed knowledge between nurses working and not working in the Pediatric Ward.

	No (n = 276)		Yes (n = 17)				95% *CI*		
	M	*SD*	*M*	*SD*	*t*	*p*	*LL*	*UL*	*d*
General knowledge	9.43	3.46	10.47	1.33	-2.72	0.011	-1.82	-0.26	0.31
Factor 1	5.63	2.58	7.06	1.39	-3.84	0.001	-2.19	-0.66	0.56
Factor 2	3.79	1.71	3.41	1.50	0.90	0.369	-0.45	1.22	0.23
Attitude	21.63	4.51	21.88	5.27	-0.22	0.825	-2.49	1.99	0.06
Self-assessment knowledge	3.18	1.06	3.18	0.95	0.02	0.986	-0.51	0.52	0.00

M – mean; SD – standard deviation; t – t-statistic; p – significance level; CI – confidence interval; LL – lower limit; UL – upper limit; d – Cohen's d effect size.
